# The impact of GeNeurofil™ nutraceutical supplementation on psychometric assessment and biomarkers of neurodegeneration in cognitive impaired patients: a pilot study

**DOI:** 10.1038/s41430-026-01791-6

**Published:** 2026-07-28

**Authors:** Luigia Trabace, Maria Grazia Morgese, Paolo Tucci, Lisa Pia Agosti, Martina Santoro, Carmen Ritrovato, Rossella Gallotto, Maurizio Tolfa, Sara Nardella, Giuseppe Cristiano, Tonia Bocci, Matteo Bevilacqua

**Affiliations:** 1 Department of Clinical and Experimental Medicine, University of Foggia, Foggia, Italy; 2 Territorial Health Center—San Marco in Lamis—Foggia Local Health Authority, Foggia, Italy; 3 Pia Gravina Foundation—San Marco in Lamis (FG), Foggia, Italy; 4 IRCCS “Casa Sollievo della Sofferenza”—S.C. Pharmacy—San Giovanni Rotondo (FG), Foggia, Italy; 5 Pharmaceutica San Marco S.r.l., Milano, Italy

**Keywords:** Drug development, Biomarkers

## Abstract

**Background/Objectives:**

Mild cognitive impairment (MCI) is characterized by a decline in cognitive functions while the ability to perform daily activities remains intact. This pathological condition is a recognized as risk factor for dementia-related disorders, and the current lack of therapies is the critical issue. Our preclinical studies indicated that a formulation (named GeNeurofil™) exhibited a synergistic neuroprotective effect in an animal model of amyloid beta toxicity. Therefore, the present study aimed to evaluate the efficacy of GeNeurofil™ in patients with mild cognitive impairment.

**Subjects/Methods:**

A six-months, randomized, double-blind, placebo-controlled pilot trial (retrospectively registered on ISRCTN registry-number ISRCTN11405046-01/07/2026) was performed enrolling 54 patients with mild to moderate MCI (Clinical Dementia Rating Scale ranging from 0.5 to 2). Patients, concurrently maintained under their usual medications, received either the GeNeurofil™ (0.7 mL × 3/day) or an identical placebo (0.7 mL × 3/day) for six months. The psychometric assessments were performed using the Mini Mental State Examination (MMSE) tests and depressive symptoms were evaluated with the Zung Test. Plasma samples were analyzed for NfL and p-TAU.

**Results:**

After six months of treatment, GeNeurofil™ supplementation improved CDR, MMSE, and Zung scores. Furthermore, the treatment reduced plasmatic levels of light neurofilaments (NfL) and phosphorylated Tau protein (p-TAU) compared to placebo.

**Conclusion:**

Our findings indicate that six months of supplementation with GeNeurofil^TM^ might represent a helpful adjuvant strategy for cognitive impairment. GeNeurofil™ nutraceutical supplementation demonstrates a favorable safety profile, however given the exploratory nature of this pilot feasibility trial, these preliminary findings will further followed by confirming definitive clinical efficacy in larger clinical trials.

## Introduction

Greater longevity is leading to increased total number of dementia cases, making it a global health burden [1, 2]. Therefore, great relevance is nowadays given to possible strategies able to prevent or even to slow down the surge of this neurological pathology.

Mild cognitive impairment (MCI) is defined as a mild impairment of cognitive function (typically memory) combined with otherwise normal functioning in daily activities. This condition lies between normal aging and Alzheimer’s disease (AD) or other forms of dementia; in fact, although patients with MCI do not necessarily develop dementia or AD, this condition is now recognized as a risk factor for AD [3]. Therefore, possible novel therapeutic strategies able to avoid or to slow down this possible transition represent an enormous challenge in basic and applied research. A critical issue is the current lack of therapies that improve the clinical course of these pathological conditions [4]. Furthermore, dementia related disorders not only are lacking of effective treatments, but many failures have been described for novel specific pharmacological approaches suggesting the involvement of other pathogenic factors, with neuroinflammation now recognized as a key target [5]. Hence, current efforts aim to identify compounds with anti-inflammatory and neuroprotective activities. This has led to the investigation of many natural products, either as novel drug candidate or as adjuvants for existing therapies approved for AD and other types of dementia. In this light, omega-3 polyunsaturated fatty acids, and in particular docosahexaenoic acid (DHA), have been shown to reduce the risk of dementia and cognitive decline [6]. Indeed, epidemiologically, individuals with dementia exhibit lower concentrations of DHA in the brain and plasma, a finding that suggests a potential therapeutic role for omega-3 polyunsaturated fatty acid supplementation [7]. On the other hand, previous studies have suggested a synergistic effect between omega-3 fatty acids and *Boswellia serrata* derivatives in pathological conditions, leading to improved quality of life [8]. In this light, *Boswellia* derivatives are considered novel candidates for neurodegenerative disorders [9]. Accordingly, our preclinical evaluations indicated that *Boswellia* derived metabolite, such as β-boswellic acid (βBA), in addition with β-caryophyllene (BCP), furanoeudesma-1,3-diene (FE) exhibited a greater and synergistic neuroprotective effect against neurodegeneration more than its individual components in an animal model of amyloid beta toxicity ([10] and patented data). Interestingly, all these substances are considered safe for human use [11].

Thus, based on these preclinical findings, we conceived this study as a pilot randomized feasibility trial, not a definitive hypothesis-testing Randomized clinical trial, however we also took the possibility of preliminary evaluating the effect of the nutraceutical (named GeNeurofil™) in patients diagnosed with mild cognitive impairment. The treatment was administered sublingually to patients with the aim of attenuating neurodegeneration and enhancing cognitive function. To evaluate the effects of the nutraceutical on neurodegeneration, plasma levels of light neurofilaments (NfL) and phosphorylated Tau protein (p-TAU) were evaluated [12–14].

## Subjects and methods

### Subjects

Fifty-four participants were enrolled at Presidio Territoriale di Assistenza (PTA), San Marco in Lamis, and Fondazione Pia Gravina, San Marco in Lamis (Foggia, Italy).

Inclusion criteria: patients aged 65–85 years with cognitive impairment ranging from mild cognitive impairment to mild/moderate dementia, as assessed by the Clinical Dementia Rating (CDR) scale (scores 0.5–2) [15].

Exclusion criteria: CDR scores <0.5 or >2, recent stroke or transient ischemic attack, unstable heart disease, renal and hepatic failure, oncologic patients, inability to provide consent or refusing to provide informed consent.

The psychometric assessments were performed by two independent raters using the Mini Mental State Examination (MMSE) [16] tests. To minimize inter-rater variability and eliminate potential scoring and administration bias, all psychometric and clinical outcome measures (including the MMSE, CDR, and Zung scales) were independently administered by two dedicated, certified clinical raters. Prior to study initiation, both raters underwent rigorous, standardized training procedures to harmonize test administration and scoring criteria. Inter-rater reliability checks were successfully performed to guarantee consistency throughout the longitudinal assessment period. Data from the Zung Test [17], which assesses depression severity, were also collected from patients’ medical records at baseline (T0) and later timepoints. Clinical assessments, questionnaires, adverse reaction reporting and biomarker measurements were performed every three months (T1 and T2). During the trial patients enrolled were kept on their routinely drug regimen.

### Ethical approval

The study entitled “Nutraceuticals in Alzheimer Disease (NAD)” was approved by the local ethics committee (Fondazione Casa Sollievo della Sofferenza di San Giovanni Rotondo, Comitato Etico Area 1 dell’Azienda Ospedaliero-Universitaria “Ospedali Riuniti” di Foggia, approval number 88/CE/2023). The study was retrospectively registered on ISRCTN registry-number ISRCTN11405046-01/07/2026. All patient data were treated confidentially and anonymously, and the study was conducted in line with the Helsinki Declaration. All patients signed for written informed consent.

### Randomization and blinding

Participants were randomly assigned in a 1:1 ratio to receive either GeNeurofil™ or Placebo. The random allocation sequence was generated a priori by an independent biostatistician using a computer-generated random number program (block randomization with variable block sizes to guarantee strict allocation concealment). The allocation sequence was concealed from the enrolling clinicians and clinical raters in sequentially numbered, opaque, sealed envelopes, which were opened only after baseline psychometric and biomarker assessments were completed.

### Treatment

GeNeurofil™ is a nutraceutical composed of:

- Clove (Syzygium aromaticum) liquid extract: 42 mg/day, of which beta-caryophyllene 37.8 mg, terpenes 4.2 mg;
- Myrrh liquid extract: 24 mg/day, of which furanodienes 19.2 mg, terpenes 4.8 mg;
- Boswellia serrata powder: 42 mg/day, of which boswellic acids 27.3 mg, amyrins 8.4 mg;
- Microalgae oil extract: 1853 mL/day, titrated 550 mg in DHA (1000 mg/day) and 3.1 mg in EPA (5.7 mg/day).
- Flavoring: lemon and sweet orange essential oils.

The placebo consisted of: edible linseed oil, flavoring (lemon and sweet orange essential oils) as above. Patients were equally allocated to the two arms according to randomization (as indicated in the CONSORT flowchart in the supplementary materials).

The nutraceutical and placebo were formulated at the Pharmacy of the “Casa Sollievo della Sofferenza”, Foggia, Italy, and administered sublingually for six months. During the study, all patients were maintained under their routine treatments.

Compliance was assessed by ampoule count at each study visit and expressed as the percentage of doses taken relative to those prescribed, with high and comparable adherence observed between groups throughout the study period.

### Study design and timepoints

The randomized, double-blind trial followed the subsequent protocol: 46 patients concluded the study and were divided into 25 subjects (mean age 76.6 years) receiving the nutraceutical preparation (0.7 mL × 3/day corresponding to 37.8 mg/day of Beta-caryophyllene, 19,2 mg/day of Furanodiene, 27.3 mg/day of boswellic acids, 1000 mg/day of Docosahexaenoic acid and 21 subjects (mean age 78 years) receiving the placebo (0.7 mL x 3/day over a six-month period.

### Plasma NfL and p-TAU measurements

Plasma samples were analyzed for NfL and human p-TAU by using ELISA kits provided by MyBioSource (San Diego, CA, USA) and Assay Genie (Dublin, Ireland), respectively. Assays were performed according to the manufacturer’s instructions. Each sample analysis was carried out in duplicate to avoid intra-assay variations.

### Statistical analysis

Statistical analysis was performed by using PRISM GraphPad 10.6 software for Windows (La Jolla, CA, USA).

Differences between groups at baseline were evaluated by Student’s unpaired t test or Fisher’s exact test for proportion data (gender and pharmacological treatments). The statistical approach used was the Intention to treat model (ITT), thus statistical evaluations between groups (placebo vs GeNeurofil™) and within group over time (T0 *vs* T1 and T2), were verified by Mixed-effects model (Restricted Maximum Likehood-REML) followed by Šídák’s multiple comparisons test. Effect size for interaction (time x GeNeurofil™) was established by calculating Cohen’s f value through G*Power3.1. A *p* value < 0.05 was considered statistically significant.

Post-hoc power analyses were performed based on observed effect sizes for all outcomes. Across this range, cognitive and biomarker outcomes demonstrated consistently high statistical power (power CDR 0.996, MMSE- > 0.999, NfL ->0.999, and p-TAU-0.964), while the Zung score remained comparatively underpowered (61%), consistent with its exploratory nature.

## Results

### Study population

**Fig. 1:**
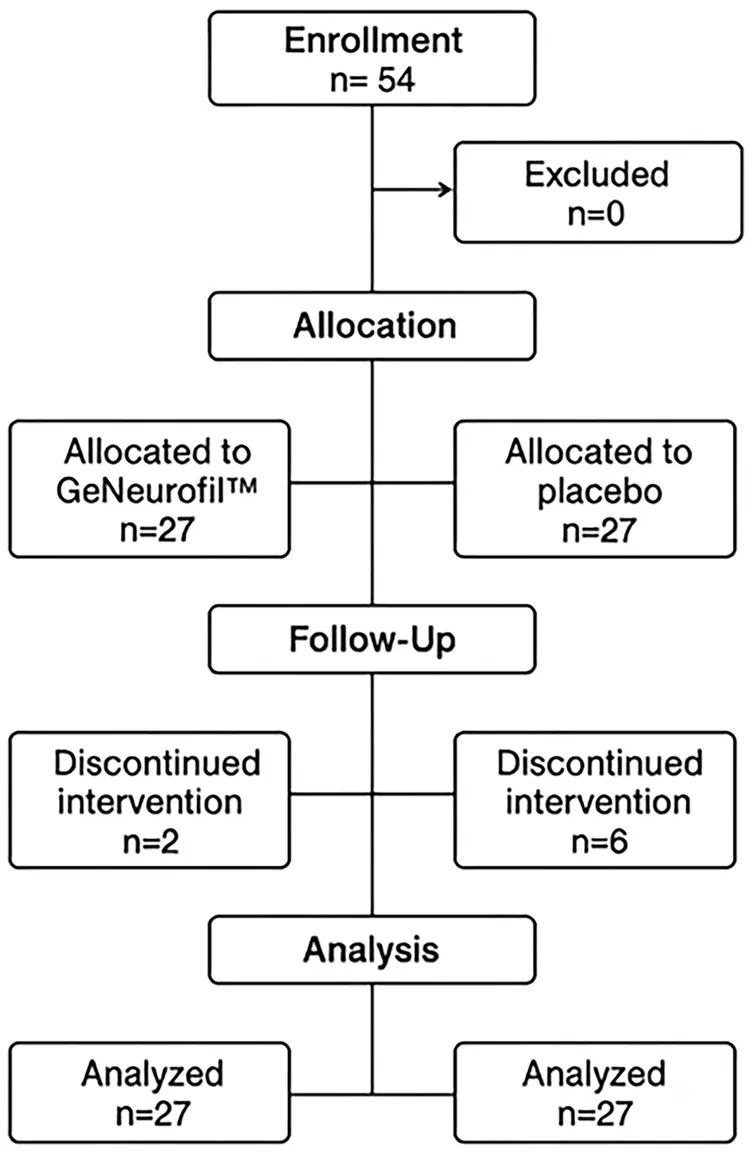
CONSORT flow diagram for GeNeurofil™ pilot study. CONSORT flow diagram of participant progress through the study stages (Enrollment, Allocation, Follow-Up, and Analysis), comparing the GeNeurofil™ group (*n* = 27) and the placebo group (*n* = 27).

A total 54 patients were enrolled in the study and were equally randomized in placebo or GeNeurofil™ group (Fig. 1), as evidences by patient characteristics detailed in Table 1. In the overall population, the percentage of female inclusion was 52%, and in particular in 43% placebo, and 59% in GeNeurofil™ (Fisher’s exact test, *P* = 0.4142). In addition, no differences were observed in baseline levels for CDR (Student’s unpaired *t* test, *P* = 0.4324), MMSE (Student’s unpaired *t* test, *P* = 0.9836), Zung (Student’s unpaired *t* test, *P* = 0.6608), and for NfL (Student’s unpaired *t* test, *P* = 0.8785) or p-TAU (Student’s unpaired *t* test, *P* = 0.7501) quantifications. The two study groups were homogeneous with respect to baseline demographic and clinical characteristics, including body mass index (BMI) and Charlson Comorbidity Index (CCI), indicating comparable frailty and comorbidity burden between the placebo and GeNeurofil^TM^-treated cohorts.

**Table 1:** Baseline characteristics of study participants^$^ and routine drug regimen (enrolled age range: 65–85 years).

Characteristic	Placebo (*n* = 27)	GeNeurofil™ (*n* = 27)	*p* value
**Demographics & anthropometrics**			
Age (years)	79.04 ± 8.6	79.18 ± 11.2	0.9622
Female sex [n (%)]	11 (40.7%)*	18 (66.7%)*	0.1007
Body Mass Index (BMI, kg/m²)	26.15 ± 4.60	27.20 ± 4.01	0.4430
**Clinical & comorbidity profile**			
Charlson Comorbidity Index (CCI)	4.20 ± 1.66	4.07 ± 1.69	0.8350
Baseline CDR	1.31 ± 0.64	1.46 ± 0.75	0.4324
Baseline MMSE	21.25 ± 3.03	21.23 ± 3.55	0.9836
Zung score	53.56 ± 13.91	55.07 ± 11.22	0.6608
**Biomarkers of neurodegeneration**			
NfL (ng/mL)	64.62 ± 10.34	64.24 ± 7.70	0.8785
p-TAU (pg/mL)	64.14 ± 26.48	62.00 ± 22.48	0.7501
**Routine drug regimen [n (%)]**			
Antihypertensives	9 (33.3%)	5 (18.5%)	0.3520
Antidepressants	3 (11.1%)	1 (3.7%)	>0.9999
Benzodiazepines	3 (11.1%)	3 (11.1%)	>0.9999
Antipsychotics	2 (7.4%)	1 (3.7%)	>0.9999
Multiple concomitant therapies†	6 (22.2%)	1 (3.7%)	0.1003

Data are presented as mean ± SD or n (%), as appropriate. Baseline differences between groups were assessed using the unpaired Student’s *t* test for continuous variables and Fisher’s exact test for categorical variables. BMI body mass index, CCI Charlson Comorbidity Index, CDR Clinical Dementia Rating, MMSE Mini-Mental State Examination, NfL neurofilament light chain, p-TAU phosphorylated tau. ^**$**^All participants were fully institutionalized inpatients with strictly standardized and controlled nutritional/dietary regimens within the clinical facility. *Note: Percentages for sex are corrected out of the group size (*n* = 27). †Multiple concomitant therapies indicate the concurrent use of two or more routine medications.

As regard to ongoing medications, Table 1 also states the type of pharmacological treatments used and poly-therapies occurring. Results showed that no statistical differences were present among the study population as revealed by Fisher’s exact test for proportions.

Eight patients (drop out from 54) discontinued for the following reasons: 2 due to fatigue (assigned to placebo), 2 due to inability to retain the preparation in the mouth (assigned to placebo), 2 due to distrust of the preparation (assigned to placebo), 1 due to family opposition (assigned to GeNeurofil™), and 1 because the taste was considered “unpleasant” (assigned to GeNeurofil™).

### GeNeurofil™ supplementation improves CDR and MMSE test performance after six months of treatment

**Fig. 2:**
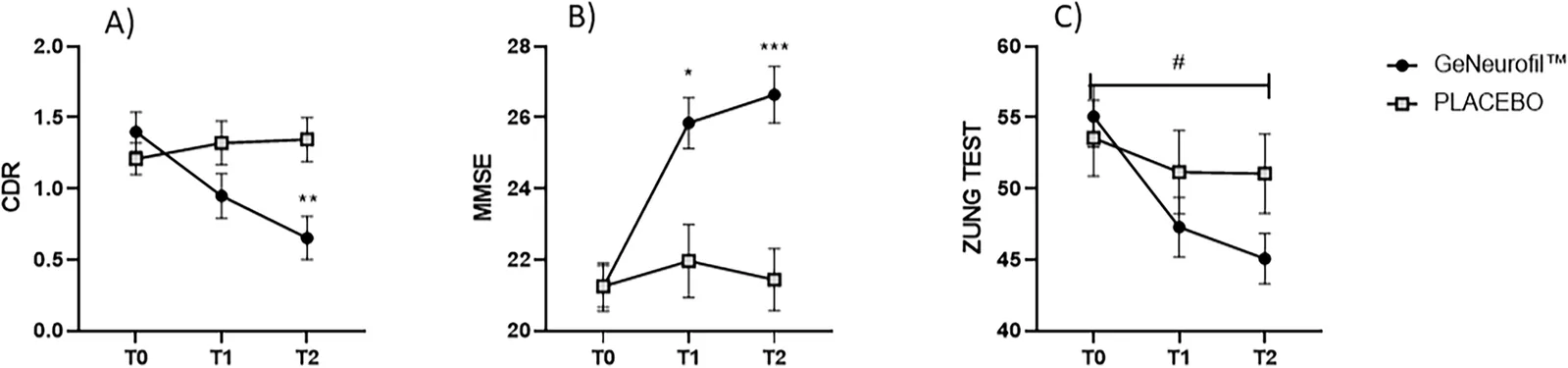
Effects of nutraceutical supplementation on clinical rating scales. The mean scores ( ± SEM) for CDR (**A**), MMSE (**B**), Zung test (**C**) in the GeNeurofil™ (black circle) and PLACEBO (gray square) groups at baseline (T0), three (T1) and six (T2) months. Significant differences between groups at different timepoints are reported: *P < 0.05; **P < 0.01; ***P < 0.001 vs PLACEBO at the same time point (Mixed-effects model- REML followed by Šídák’s multiple comparisons test). Zung test: significant differences across time; (REML), # *P* < 0.05.

After six months of treatment (T2), psychometric cognitive assessments showed significant improvements in the GeNeurofil™ group compared to the placebo group. In particular, for the CDR score, REML analysis revealed a significant effect in the interaction between time and GeNeurofil™ treatment (Fig. 2A; REML: time x GeNeurofil™= F (2, 87) = 6553, *P* = 0.022, Multiple comparisons with post-hoc Šídák’s test: T2 *P* < 0.01); while no statistical significance was reached for main effect of both time and treatment (time = F _(1.171, 50.96)_ = 3403 *P* = 0.0646 and GeNeurofil™ F (1, 52) = 3.936, *P* = 0.0525). Moreover, the MMSE score was overtime increased in GeNeurofil™ -treated patients (Fig. 2B; REML: time x GeNeurofil™= F (2, 88) = 9.474, *P* = 0,0002 Multiple comparisons with post-hoc Šídák’s test: T1 *P* < 0.05 GeNeurofil™ *vs* placebo, and T2 GeNeurofil™ *vs* placebo *P* < 0.001; time F _(1.233, 54.27)_ = 11.85, *P* = 0.0005, GeNeurofil™ F (1, 52) = 15.97 *P* = 0.0002). The analysis of Zung test score showed only significant reduction across timepoint score independently from treatment (Fig. 2C; REML, F _(1.196, 49.62)_ = 4439, *P* = 0.0337). No statistical differences were evidenced for GeNeurofil™ nor for interaction between time and treatment (REML: time x GeNeurofil™= F (2, 83) = 1.500, *P* = 0.2292; GeNeurofil™ F (1, 52) = 1.717, *P* = 0.1948). Effect size for these scores were indicated as following: f_*CDR*_ = 0.3881, f_*MMSE*_ = 0.4641, f_*Zung test*_ = 0.1904.

### GeNeurofil™ supplementation reduces plasmatic levels of NfL and p-TAU after six months of treatment

**Fig. 3:**
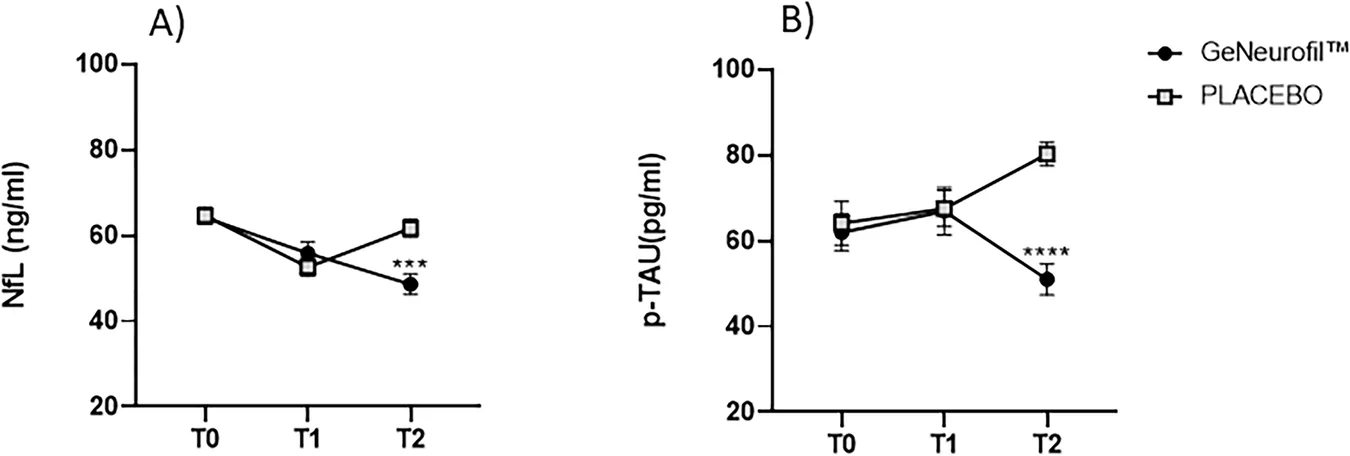
Effect of nutraceutical intervention on plasma biomarkers of neurodegeneration. The mean value (± S.E.M.) in plasma concentrations of NfL (**A**, ng/ml) and p-TAU (**B**, pg/ml) for the GeNeurofil™ (black circle) and PLACEBO (gray square) groups at baseline (T0), three (T1) and six (T2) months. Significant differences between the two groups were determined using mixed effect model REML followed by Šídák’s multiple comparisons test)T2: *** *P* < 0.001 and **** *P* < 0.0001for NfL and p-TAU, respectively.

The GeNeurofil™ treatment caused a significant decrease compared to the placebo group in plasma NfL levels over time (Fig. 3A; REML: time x GeNeurofil™= F (2, 81) = 7.854, *P* = 0.0008, time F _(1.972, 79.86)_ = 15.84, *P* < 0.0001; Multiple comparisons with post hoc Šídák’s test: T2 *P* < 0.001); no statistical significance was reached for main effect of treatment (GeNeurofil™ F (1, 52) = 3.527, *P* = 0.0660). P-TAU levels followed the same pattern with a significant reduction in plasma levels of p-TAU at T2 in patients receiving GeNeurofil™ (Fig. 3B; REML: time x GeNeurofil™ = F (2, 133) = 5.600, *P* = 0.0046; Multiple comparisons with post-hoc Šídák’s test: T2 *P* < 0.0001 GeNeurofil™ vs placebo; F time _(1.408, 93.63)_ = 0 4638, *P* = 0.5626, F GeNeurofil™ _(1, 133)_ = 7.991, *P* = 0.0054). Effect size for these scores were indicated as following: f_*NfL*_ = 0.4403, f_*p-TAU*_ = 0.2909.

### Safety evaluation of GeNeurofil™ supplementation

A total of 8 mild AEs were recorded throughout the 6-month study period, all of which led to study discontinuation. Placebo Group (*n* = 27): 6 AEs were reported. These consisted of fatigue (*n* = 2; 7.4%) and transient oral/palatability discomfort described as the inability to retain the preparation in the mouth (*n* = 4; 14.8%). GeNeurofil™ Group (*n* = 27): 2 AEs were reported, both consisting of mild palatability discomfort described as an unpleasant taste (*n* = 2; 7.4%). The remaining discontinuation in this group was due to family opposition, which was classified as a non-safety withdrawal rather than an AE. All 8 recorded AEs were classified as mild in severity. Critically, zero (0) AEs were deemed to have a probable or definite causal relationship with the active ingredients of GeNeurofil™. The fatigue reported by 2 participants occurred exclusively within the placebo cohort. Statistical Comparison: The overall incidence of AEs did not differ significantly between the groups (22.2% in the Placebo group vs. 7.4% in the GeNeurofil™ group; Fisher’s exact test, *p* = 0.2471), confirming a comparable safety and tolerability profile.

## Discussion

In the present pilot study, we tested the effect of the nutraceutical GeNeurofil™ on several psychometric assays in patients affected by mild dementia or cognitive impairment. According the results obtained, the nutraceutical GeNeurofil™ was able to reduce CDR and to increase the MMSE scores in the treated group, along with a reduction in two biomarkers of neurodegeneration.

The dementia and AD are characterized by a long preclinical period and patients typically show the first signs of impairment when certain brain areas exhibit a significant level of neurodegeneration, with the appearance of beta amyloid deposition and tau tangles. During this clinical window, these processes are self-propagating and the disease usually progresses rapidly. Thus, we can speculate that GeNeurofil™ might slow down disease progression by acting on neurodegenerative processes, as suggested by reduction in plasma NfL and p-TAU levels obtained after six months of treatment.

The NfL protein is considered a product of neuroaxonal degeneration released into the cerebrospinal fluid and subsequently into the bloodstream. Indeed, its plasmatic levels increase in several neurodegenerative diseases, such as Parkinson’s disease, amyotrophic lateral sclerosis and other neurological disorders.

The p-TAU plasmatic levels demonstrate high specificity for AD. These levels correlate directly with the beta amyloid load and tau tangles in the brain. However, plasma p-TAU can be detected in the preclinical phase, predicting the conversion from MCI to AD. Among the nutraceutical GeNeurofil™ ingredients, βBA interacts with the TAU protein, forming a stable βBA-TAU complex through hydrophobic bonds and stabilizing microtubules. Furthermore, it has been shown to reduce the hyperphosphorylation of TAU protein and amyloid-beta levels, leading to improved learning and memory. It affects the polymerization kinetics of microtubules and consequently the axonal growth and neurite branching of hippocampal neurons [18].

Interestingly, the results of this pilot study indicate that the nutraceutical in addition to slowing disease progression, has enhanced the cognitive skills, as suggested by an increased score on the MMSE and by the decrease in CDR. βBA has been shown to improve memory by acting on the expression of the CREB-1 and CREB-2 genes [19]. Furthermore, it induces neuritogenesis [20] while BCP, another active the component of GeNeurofil^TM^ induces synaptogenesis by the upregulation of axonal plasticity-related proteins (GAP-43, synapsin, and synaptophysin) and the activation of the neurotrophic receptor trkA, a member of the neurotrophic tyrosine kinase receptor family [21].

Although not statistically significant, it should be noted that the proportion of participants receiving multiple concomitant therapies was higher in the placebo group compared to the GeNeurofil™ group. This imbalance in baseline polypharmacy may represent a potential confounding factor, as concomitant treatments could influence cognitive trajectories in our study population and should therefore be considered when interpreting the present findings.

Therefore, we observed a reduction in the Zung score in patients receiving both placebo and GeNeurofil™. This effect is actually quite common in nutraceutical trials for depression and several predisposing factors have been individuated for explaining it, such as placebo effects or even therapeutic alliance [22]. A recent meta-analysis that included a series of randomized clinical trials of antidepressants from the last 30 years highlighted that since 1980 there has been a steady increase in the effect of placebo on depression scores, which may explain the apparent decline in the efficacy of antidepressants in clinical trials in recent years [23]. Nevertheless, we cannot rule out the possibility that the cognitive improvement in treated patients might have contributed to Zung score reduction as well.

Depression is a typical comorbidity in AD patients and recognized as one of the 14 risk factors for dementia [24]. Depression represents a risk factor across the adult lifespan, in addition to being a potential prodromal stage of AD [25–29].

This outcome seems confirming what we already demonstrated in an animal model of amyloid beta-induced toxicity, where the *Boswellia* derivative βBA was able to reduce depressive-like symptoms and to restore the alteration in levels of neurotransmitters and neurotrophins [10]. Additionally, βBA counteracts the depressive-like phenotype induced by the intracerebroventricular injection of beta amyloid in rodents in a way that resembles what we have also demonstrated with omega-3 fatty acids supplementation [10, 30]. In the same animal models, the mixture of these substances demonstrated greater antidepressant effects than its individual components administered alone (patented data). It should be noted that also placebo groups showed the same pattern indicating a possible placebo response.

### Study limitations

Several limitations must be acknowledged when interpreting the findings of this pilot trial. First, the study population is characterized by an advanced mean age (79 years), which reflects a real-world institutionalized cohort but may limit the generalizability of the findings to younger populations experiencing early cognitive decline. Second, despite the lack of a statistically significant difference at baseline, the higher proportion of multiple concomitant therapies in the placebo group (22.2%) compared to the GeNeurofil™ group (3.7%) represents a potential confounding factor that may independently influence longitudinal cognitive trajectories. Lastly, as an exploratory pilot feasibility study, this trial was not primarily powered for broad efficacy conclusions.

## Conclusions

In conclusion, our findings provide the first clinical evidence that six months of GeNeurofil™ supplementation, when administered as an adjunct to standard pharmacological treatment, may improve cognitive performance in patients with mild-to-moderate Alzheimer’s disease. These findings warrant further investigation in larger, randomized controlled trials and support the potential of nutraceutical supplementation as a safe, accessible, and targeted strategy to help preserve cognitive function.

## Supplementary information

suppementary materials consort checklist

## Data Availability

All raw data will be made available to interested scientists upon reasonable request, subject to data privacy regulations and proprietary information agreements, as detailed in the paper’s methods section. Furthermore, we guarantee full commitment to sharing the full anonymized clinical and biomarker dataset with independent researchers for verification purposes, while noting that only data related to the proprietary chemical composition beyond what is listed is withheld.
