# Supplementary material for: The impact of GeNeurofil™ nutraceutical supplementation on psychometric assessment and biomarkers of neurodegeneration in cognitive impaired patients: a pilot study
Source: Eur J Clin Nutr. 2026 Jul 28;80(9):943–8. doi: 10.1038/s41430-026-01791-6 (PMC13558051; doi:10.1038/s41430-026-01791-6)
Supplement: Supplementary file 1 [file 41430_2026_1791_MOESM1_ESM.docx]

**CONSORT 2010 Checklist**

*Trial Reporting Checklist - GeNeurofil™ Study*

| **Section / Topic** | **Item No** | **CONSORT Checklist Item** | **Reported in Manuscript / Study Details** |
| --- | --- | --- | --- |
| Title & Abstract | 1a | Identification as a randomized trial in the title. | Yes. The title explicitly includes 'A Pilot Study' and 'Randomized Feasibility Trial'. |
| Title & Abstract | 1b | Structured summary of trial design, methods, results, and conclusions. | Yes. The Abstract has been revised to explicitly state that this is a 'pilot study'. |
| Introduction | 2a | Scientific background and explanation of rationale. | Yes. Explains the impact of GeNeurofil™ nutraceutical supplementation on cognitive trajectories and neurodegeneration biomarkers. |
| Introduction | 2b | Specific objectives or hypotheses. | Yes. Exploratory pilot evaluation to inform the design of future, larger clinical trials. |
| Methods - Trial Design | 3a | Description of trial design (such as parallel, factorial) including allocation ratio. | Parallel groups. Equal allocation (1:1 ratio) at randomization. |
| Methods - Trial Design | 3b | Important changes to methods after trial commencement (such as eligibility criteria), with reasons. | None. Inclusion criteria approved by the Ethics Committee were strictly followed during the study's execution. |
| Methods - Participants | 4a | Eligibility criteria for participants. | Yes. Patients aged 65–85 years with cognitive impairment. Inpatients with fully controlled nutritional intake within the clinical setting. |
| Methods - Participants | 4b | Settings and locations where the data were collected. | Yes. Clinical setting (inpatients). |
| Methods - Interventions | 5 | The interventions for each group with sufficient details to allow replication, including how and when they were actually administered. | Yes. GeNeurofil™ Nutraceutical Supplementation vs. Placebo (Control). |
| Methods - Outcomes | 6a | Completely defined pre-specified primary and secondary outcome measures, including how and when they were assessed. | Yes. Cognitive measures (CDR, MMSE, Zung) and biomarkers of neurodegeneration (NfL, p-TAU). |
| Methods - Sample Size | 7a | How sample size was determined. | Pilot Framework. Formal a priori sample size calculation not strictly required; designed to gather preliminary data and observe baseline effect sizes. |
| Randomization | 8a | Method used to generate the random allocation sequence. | Yes. The text clarifies and specifies the method used to generate the random sequence. |
| Randomization | 9 | Mechanism used to implement the random allocation sequence, describing any steps taken to conceal the sequence until interventions were assigned. | Yes. Described within the methodology section. |
| Randomization | 10 | Who generated the random allocation sequence, who enrolled participants, and who assigned participants to interventions. | Yes. Included in the trial protocol description. |
| Blinding | 11a | If done, who was blinded after assignment to interventions and how. | Double-blinded framework. Evaluated via two independent, trained raters following standardized procedures to minimize scoring and administration bias. |
| Statistical Methods | 12a | Statistical methods used to compare groups for primary and secondary outcomes. | Yes. Linear mixed-effects models estimated by Restricted Maximum Likelihood (REML). All n=54 randomized participants are preserved in the model. |
| Statistical Methods | 12b | Methods for additional analyses, such as subgroup analyses and adjusted analyses. | Yes. Sensitivity-based post-hoc power analysis using real non-sphericity epsilon (ε) values, and post-hoc baseline homogeneity checks (BMI, CCI). |
| Results - Participant Flow | 13a | Number of participants who were randomly assigned, received intended treatment, and were analyzed for the primary outcome. | Yes (See CONSORT Diagram): Enrolled/Allocated: n=54 total (n=27 GeNeurofil™, n=27 Placebo). Analyzed: n=46 total (n=25 GeNeurofil™, n=21 Placebo). |
| Results - Participant Flow | 13b | Losses and exclusions after randomization, together with reasons. | Yes (See CONSORT Diagram): GeNeurofil™ group (n=2 discontinued: 1 due to unpleasant taste, 1 due to family opposition). Placebo group (n=6 discontinued: 2 due to fatigue, 2 due to inability to retain preparation in mouth, 2 due to distrust of preparation). |
| Results - Baseline Data | 14a | A table showing baseline demographic and clinical characteristics for each group. | Yes. Table 1 included (Mean age ~79 years, BMI, Charlson Comorbidity Index, multi-therapies). |
| Results - Numbers Analyzed | 16 | Number of participants (denominator) included in each analysis and whether the analysis was by original assigned groups. | Yes. Intention-to-Treat (ITT) population utilized (n=54). REML estimation preserved all 54 randomized subjects by utilizing all existing observations rather than listwise deletion. |
| Results - Outcomes | 17a | For each primary and secondary outcome, results for each group, and the estimated effect size and its precision. | Yes. Reported effect sizes: f(CDR)=0.3881, f(MMSE)=0.4641, NfL, p-TAU, etc. |
| Results - Harms | 19 | All important harms or unintended effects in each group. | Yes. No serious or treatment-related adverse events reported for GeNeurofil™. Documented tolerability-related complaints leading to drop-out (fatigue, taste). |
| Discussion - Limitations | 20 | Trial limitations, addressing sources of potential bias, imprecision, and, if relevant, multiplicity of analyses. | Yes. Discussed baseline polypharmacy, advanced mean age (79 years) limiting younger generalizability, and exploratory endpoints. |
| Discussion | 21 | Generalizability (external validity, applicability) of the trial findings. | Yes. Reflects target population of elderly patients with cognitive impairment routinely observed in clinical practice. |
| Discussion | 22 | Interpretation consistent with results, balancing benefits and harms, and considering other relevant evidence. | Yes. Conclusions tempered to state that GeNeurofil™ 'shows potential to provide cognitive benefits' to better reflect the exploratory nature of the trial. |
| Other Information | 24 | Where the full trial protocol can be accessed, if available. | Yes. Approved by the local Ethics Committee. |
